# Special reviews on the history and future of the Korean Institute of Medical Education and Evaluation to memorialize its collaboration with the Korea Health Personnel Licensing Examination Institute to designate JEEHP as a co-official journal

**DOI:** 10.3352/jeehp.2020.17.33

**Published:** 2020-10-22

**Authors:** Sun Huh

**Affiliations:** Department of Parasitology and Institute of Medical Education, College of Medicine, Hallym University, Chuncheon, Korea

On June 17, 2020, a ceremony was held at Jinpungjeong (進豊呈), a Chinese restaurant in downtown Seoul (Figs. 1, 2), to mark the agreement between the Korea Health Personnel Licensing Examination Institute (KHPLEI) and the Korean Institute of Medical Education and Evaluation (KIMEE) to designate *Journal of Educational Evaluation for Health Professions* (JEEHP)―the institutional journal of the Korea Health Personnel Licensing Examination Institute―as a co-official journal of the KIMEE. This step was taken to encourage researchers who work in the field of medical school accreditation to submit their study results to JEEHP.

This editorial aims to briefly introduce the role of KIMEE and the content of 4 relevant reviews that have been published in JEEHP. These materials will be helpful for medical educators who are interested in their countries’ accreditation body.

## Two essential organizations for medical education in Korea: the KHPLEI and KIMEE

The KHPLEI, which was launched in 1992 with the name of the “Korea Medical Licensing Examination Board,” and the KIMEE, which was established in 1998 with the name of the “Accreditation Board for Medical Education in Korea,” have been the most influential organizations in the context of medical education and management of medical schools in Korea. The KHPLEI has announced the content of a written test based on physicians’ competency. It has also announced the goal of implementing a clinical skill examination based on the minimal competency of a primary care physician, including core clinical presentation. All medical faculty members in Korea should be alert to information released by the KHPLEI not to miss any content regarding both the written test and clinical skill examination of the Korea Medical Licensing Examination.

The medical school accreditation programs implemented by the KIMEE have also been a potent tool for improving the educational system to the international level. I remember that the Korean Council for University Education conducted a medical school evaluation in 1996. As a junior faculty member of a medical college, I also participated in preparing for the evaluation. A report was published, and specialists visited the college to check whether the report contained accurate descriptions. At that time, the faculty members and visitors met and discussed strategies for further development in medical education. Although this was only the first step in Korea towards the accreditation of professional education, including the medical field, I was impressed by the evaluation items and the devotion of the faculty members of my medical college. This was the first time that I had the opportunity to systemically summarize the mission, vision, and goals of medical education and to look at the curriculum precisely. The experience of that evaluation process helped me to execute the role of Vice-Dean for Education of my college from February 1999 to August 2000. During the consecutive accreditation processes of the 1st cycle (2000–2005), 2nd cycle (2007–2010), and post-2nd cycle (2012–2018), the dean of my medical school was able to obtain budgetary resources for upgraded facilities, including a team-based learning room, from the headquarters of the university.

## Four reviews on the KIMEE translated from Korean

I would like to introduce 4 reviews of the history, activities, and future of the KIMEE to memorialize its collaboration with the KHPLEI for official journal publishing. Those 4 reviews were already published in Korean in the *Korean Medical Education Review* (pISSN 2092-5603, eISSN 2093-6370). I asked Dr. Woo Taek Jeon, editor of the *Korean Medical Education Review*, to allow me to publish these reviews in JEEHP as secondary publications. All authors of the 4 reviews allowed this. I translated the 4 reviews into English. The authors confirmed the translated versions, in which some modifications occurred according to the journal’s style and format.

“History of the medical education accreditation system in Korea: implementation and activities in the early stages” by Dr. Kwang-ho Meng is a vivid presentation on the early stage of the KIMEE’s activities [1]. Dr. Meng is not only a pioneering epidemiologist, but also a specialist in medical education in Korea. He has published dozens of articles on medical education, and had worked for the KIMEE on a volunteer basis. He is one of the professors who observed medical education accreditation in Korea from its early stages. From his review, I learned about the gift of foresight of the late Dr. Yoo Bock Lee (1927–2018), who was a professor of pathology at Yonsei University College of Medicine. Dr. Lee argued for the necessity of an accreditation system for medical education and graduate medical education in Korea in his article published in 1990 [2]. His ideas were realized later by the establishment of the KIMEE for medical education accreditation. Dr. Meng’s review provides valuable insights into how the pioneers in this field strived to establish a medical education accreditation system.

The second review was written by Dr. Ducksun Ahn, emeritus professor of Korea University [3]. He still works as the director of the Korea Medical Association Research Institute of Health Care Policy (2018–). He served as the president of the Western Pacific Association for Medical Education (formerly Association for Medical Education in the Western Pacific Region) from 2010 to 2014. Furthermore, during his work as vice-president of the World Federation of Medical Education (WFME, 2015–2023), he played a crucial role in hosting the WFME 2019 conference in Korea from April 7 to April 10, 2019. He was also a pioneer in introducing the medical education accreditation system in Korea. His review on global trends in accreditation for basic medical education discusses the need to promote accreditation for future medical services that will be needed in an aging society, the intensification of health systems science, and changes in the role of emergency medicine. He stressed 2 goals for future work: quality assurance and quality improvement in medical education. This work will serve as excellent food for thought for the next generation of medical educators.

The third review was written by 3 authors: Drs. Hanna Jung, Woo Taek Jeon, and Shinki An at the Yonsei University College of Medicine. They propose that medical education accreditation has pedagogic value [4], and mention 3 kinds of value: quality and equity, efficiency, and choice. They state, “Therefore, it is necessary to consider how the quality of medical education can be effectively improved while considering equity and excellence in medical school accreditation. Efficiency can be improved by revising and formalizing procedures. The value of choice in medical school accreditation is multi-faceted so that graduates can move to other countries. Nonetheless, the results of accreditation may harm schools’ reputation.” They also suggest methods to actualize the educational value of accreditation, including collaborative efforts between the accreditation body and medical schools, regular review of standards and sharing of clear guidelines, and the implementation of for formative evaluation activities.

The fourth review was written by Dr. Ki-Young Lim, who worked for the KIMEE as the director of the Accreditation Board of Medical Education in Korea from March 2010 to February 2013. He also served as the president of the Korean Society of Medical Education from January 2017 to December 2018. He proposed directions for medical education accreditation in Korea, emphasizing glocalization, the importance of interprofessional education and health systems science, and the necessity for accreditation to encompass the entire process of medical education, ranging from basic medical education to postgraduate medical education and continuing professional education [5]. I believe that his insights into the future of medical education will be important to keep in mind. It will also be necessary to incorporate these topics more actively into the curriculum and the accreditation policy.

## JEEHP is open to other organizations as a co-official journal

Cooperation between 2 essential institutes for the evaluation and accreditation of medical education in Korea will be a major step forward for enhancing the status of JEEHP as a highly informative journal on the global stage. JEEHP is a unique scholarly journal in terms of its aims and scope. JEEHP became a co-official journal of the Western Pacific Association for Medical Education in 2012, and has been an affiliated journal of the World Federation for Medical Education since September 2016 [6]. Thus, this is its 3rd agreement with other related organizations. JEEHP is open for collaboration with other related organizations throughout the world, especially in the fields of licensing examinations and accreditation.

## Figures and Tables

**Fig. 1.**
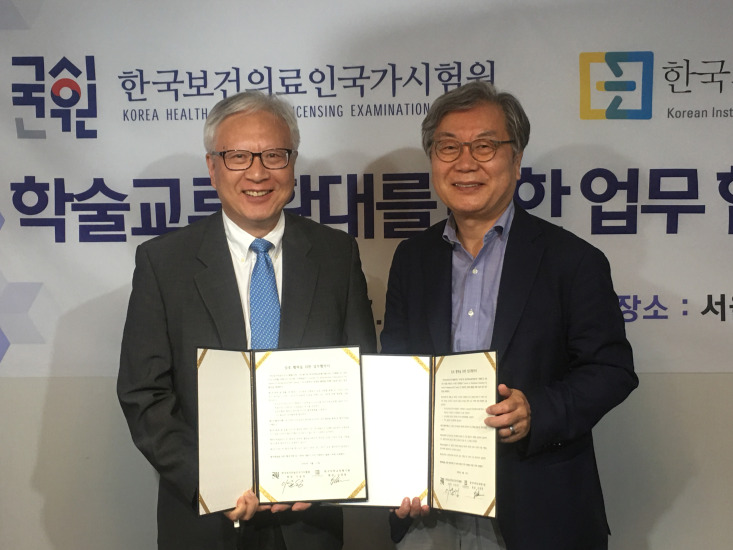
Photo of the ceremony at Jinpungjeong (進豊呈), a Chinese restaurant in downtown Seoul, Korea, to mark the agreement between the KHPLEI and the KIMEE to designate *Journal of Educational Evaluation for Health Professions* as a co-official journal of the KIMEE. Right leteral to left lateral: Dr. Yoon-Seong Lee, President of the KHPLEI and Dr. Young Chang Kim, President of the KIMEE. KHPLEI, Korea Health Personnel Licensing Examination Institute; KIMEE, Korean Institute of Medical Education and Evaluation.

**Fig. 2.**
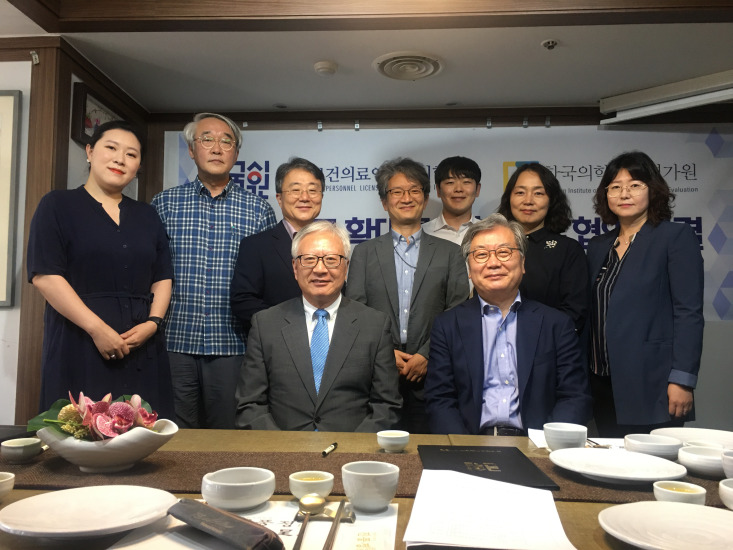
Photo of the ceremony at Jinpungjeong (進豊呈), a Chinese restaurant in downtown Seoul, Korea, to mark the agreement between the KHPLEI and the KIMEE to designate *Journal of Educational Evaluation for Health Professions* as a co-official journal of the KIMEE. Right latere to left lateral: Jushin Youn, staff of KIMEE; Duck Sun Ahn, Yonsei University, Senior Vice-President of KIMEE; Tai Young Yoon, Kyung Hee University, Vice-President of KIMEE; Yoon-Seong Lee, President of KHPLEI; Sun Huh, Hallym University, Editor; Joon Ki Kim, Research and Development Bureau, KHPLEI; Young Chang Kim, Soonchunhyang University, President of KIMEE; Kyoungsin Lee, Research and Development Bureau, KHPLEI; and Mi Kyoung Yim, Research and Development Bureau, KHPLEI. KHPLEI, Korea Health Personnel Licensing Examination Institute; KIMEE, Korean Institute of Medical Education and Evaluation.
